# Are homeostatic mechanisms aiding the reconstitution of the T-cell pool during lymphopenia in humans?

**DOI:** 10.3389/fimmu.2022.1059481

**Published:** 2022-11-22

**Authors:** Mariona Baliu-Piqué, Kiki Tesselaar, José A. M. Borghans

**Affiliations:** Center for Translational Immunology, University Medical Center Utrecht, Utrecht, Netherlands

**Keywords:** T-cell reconstitution, hematopoietic stem cell transplantation (HSCT), homeostatic mechanisms, lymphopenia, homeostatic regulation

## Abstract

A timely recovery of T-cell numbers following haematopoietic stem-cell transplantation (HSCT) is essential for preventing complications, such as increased risk of infection and disease relapse. In analogy to the occurrence of lymphopenia-induced proliferation in mice, T-cell dynamics in humans are thought to be homeostatically regulated in a cell density-dependent manner. The idea is that T cells divide faster and/or live longer when T-cell numbers are low, thereby helping the reconstitution of the T-cell pool. T-cell reconstitution after HSCT is, however, known to occur notoriously slowly. In fact, the evidence for the existence of homeostatic mechanisms in humans is quite ambiguous, since lymphopenia is often associated with infectious complications and immune activation, which confound the study of homeostatic regulation. This calls into question whether homeostatic mechanisms aid the reconstitution of the T-cell pool during lymphopenia in humans. Here we review the changes in T-cell dynamics in different situations of T-cell deficiency in humans, including the early development of the immune system after birth, healthy ageing, HIV infection, thymectomy and hematopoietic stem cell transplantation (HSCT). We discuss to what extent these changes in T-cell dynamics are a side-effect of increased immune activation during lymphopenia, and to what extent they truly reflect homeostatic mechanisms.

## Introduction

In homeostasis, lymphocyte numbers remain relatively constant thanks to a delicate balance between lymphocyte production and loss. This balance is key to a proper functioning of the immune system and gets brutally disturbed in individuals undergoing hematopoietic stem cell transplantation (HSCT). The combination of chemotherapy and/or total body irradiation and HSCT is an increasingly common treatment for many types of malignancies and immune disorders. Alemtuzumab (anti-CD52) and anti-thymocyte globulin (ATG) are two immune-suppressive antibodies that are broadly used as conditioning regimen for HSCT and are directed against T cells and non-T cells, including B cells and NK cells (1–4). Following HSCT, alemtuzumab and ATG treatment, there is a quick decline in total lymphocyte and CD3^+^ T-cell numbers, after which the body enters a long phase of immune reconstitution. Preventing lymphopenia-related complications, such as opportunistic infections and viral reactivation, critically depends on the recovery of immune cells. Indeed, timely reconstitution of the lymphocyte pool post-HSCT is clearly associated with a reduced risk of infectious complications and disease relapse (5, 6).

How lymphocyte production and loss are regulated when homeostasis gets disturbed has been a subject of both great interest and debate. Most knowledge on homeostasis (see **BOX 1** for ambiguities about the term homeostasis) and homeostatic responses to low cell numbers comes from experiments in mice. There is convincing evidence in mice that lymphocyte production and survival are homeostatically increased following severe lymphopenia (8–10). Based on this, it is generally thought that T-cell homeostasis is an active process in which new T cells are continuously produced and are competing with pre-existing cells for proliferation or survival factors (for an in-depth review on mouse T-cell homeostasis, see (8)). Theoretically, this should result in increased lymphocyte production rates and/or decreased cell loss rates when cell numbers are low. In mice, the earliest evidence for this concept dates back to 1984, based on the observation that T cells adoptively transferred into a lympho-depleted or thymectomized host quickly expand in numbers, through a phenomenon termed lymphopenia-induced proliferation (9). The occurrence of lymphopenia-induced T-cell proliferation in mice suggests that the immune system has an intrinsic capacity to quickly return to steady-state cell numbers by increasing the rate at which T cells proliferate.

Box 1 **Definition of homeostasis and homeostatic regulation.**

Homeostasis is the steady condition of a living organism. T-cell homeostasis can be defined as a means to “keep useful T cells alive and live T cells useful” (7). Homeostatic regulation was originally described as the ‘‘return tendency, due to a density-dependent process to approach a stationary distribution of population densities’’ (8). The confusion on the term *homeostasis* began when *homeostatic proliferation* started to be used to describe the slow rate of cell division that maintains lymphocyte populations in steady state, rather than the process in which T-cell division rates are increased when cell numbers are low. Here we refer to homeostatic regulation as the control of population densities, i.e. lymphocyte numbers, based on lymphocyte competition for limiting resources and survival signals. We conceive homeostatic responses to lymphopenia as a compensatory mechanism, distinct from a conventional immune response, to respond to low cell numbers by regulating cell production and loss in a cell density-dependent manner.

Unfortunately, mice are not representative of all relevant aspects of the human immune system, and there are important limitations in extrapolating findings from mice to humans (11, 12). We have previously shown that humans and mice differ fundamentally in the way naive T cells are maintained. While in mice, new naive T cells are almost exclusively produced by the thymus, in humans, peripheral proliferation is the dominant mechanism of naive T-cell production during adult life (13). Moreover, laboratory mice are typically kept in very clean circumstances, which impact the dynamics of T cells in a way that may not be representative for the human situation (14, 15). In fact, lymphocyte reconstitution in humans is known to occur notoriously slowly. Total lymphocyte and CD3^+^ T-cell numbers remain low for at least one year after immune-depletion induced by treatments such as alemtuzumab, ATG or HSCT (16–20). CD4^+^ T-cell reconstitution is particularly slow, typically taking months to years (21, 22). Other typical features of T-cell reconstitution in these settings are the slow recovery of naive T cells, and in some cases an inversion of the CD4:CD8 ratio, caused by a relatively early recovery of the CD8^+^ T-cell subset, which can persist for years (16, 18, 22–27). Taken together, this raises the question whether the homeostatic mechanisms that have been observed in mice also occur in humans and help the reconstitution of the T-cell pool after HSCT.

Studying the homeostatic response to lymphopenia after HSCT in humans is not straight-forward, as it is easily confounded by different external factors, such as opportunistic infections, graft versus host disease (GVHD) and therapy. Here, we review evidence for and against the existence of homeostatic compensatory mechanisms to low T-cell numbers in humans in a wide variety of situations in which lymphocyte numbers are low, including the early development of the immune system, healthy ageing, HIV infection, thymectomy and HSCT. We discuss to what extent the observed changes in T-cell dynamics in these specific situations can be attributed to the side effects of increased immune activation during lymphopenia, and to what extent they truly reflect a lymphopenia-driven homeostatic response that aids immune reconstitution.

## Possible mechanisms of homeostatic response to lymphopenia

**Figure 1:**
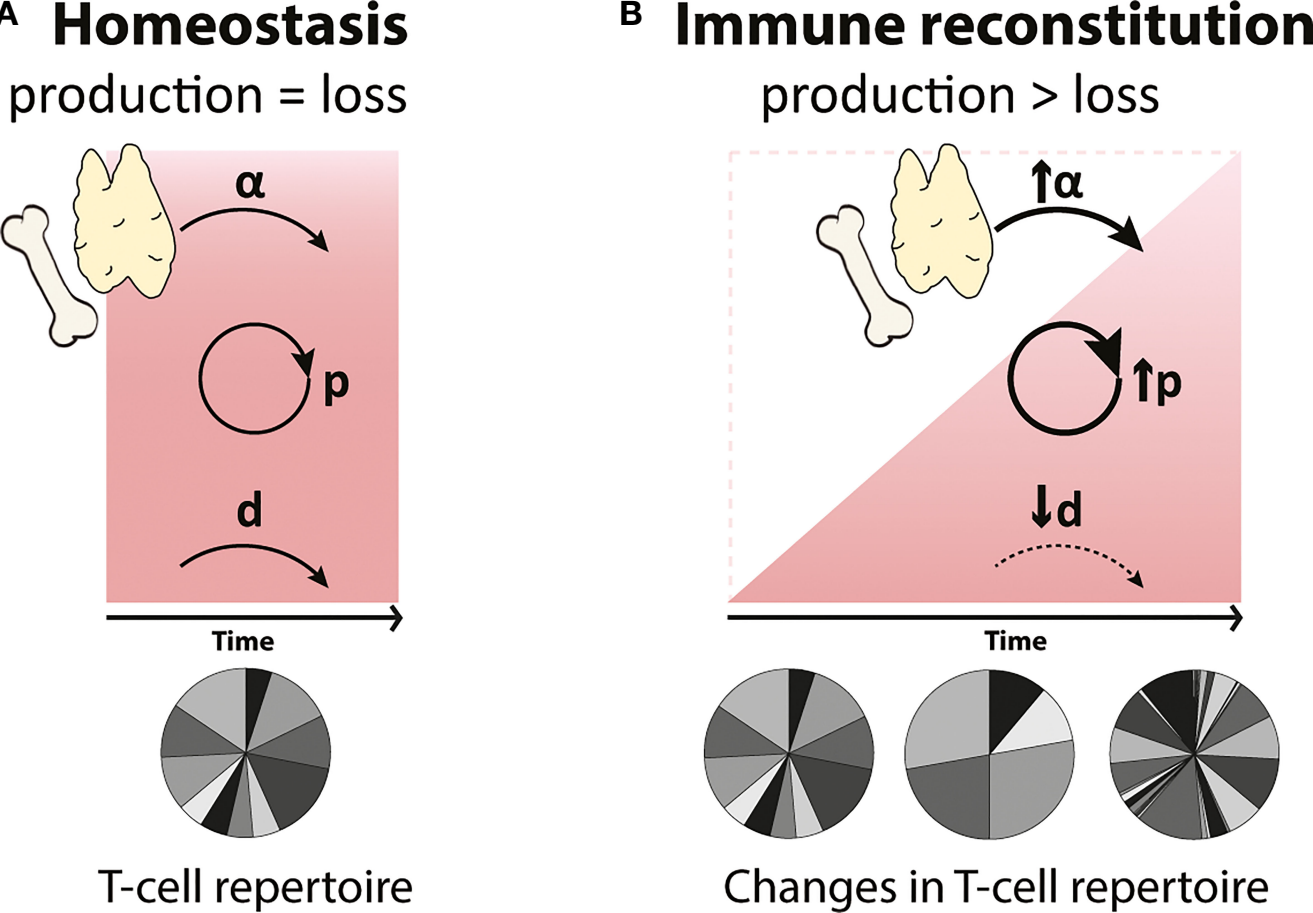
Possible compensatory mechanisms governing T-cell reconstitution during lymphopenia **(A)** In homeostasis, T-cell numbers (represented by the red square) are maintained at relatively constant levels by the balanced generation and loss of T cells. T cells are generated by thymic output (α) and by T-cell proliferation (p), and these processes are balanced by the loss of T cells (d) through cell death, maturation and migration. **(B)** During immune reconstitution (represented by the red triangle), changes in *de novo* production (α), proliferation (p) and loss (d) rates of T cells may occur to compensate for low T-cell numbers. Increased peripheral T-cell proliferation (↑p) will lead to a faster reconstitution but may generate a more clonal T-cell repertoire. An increase in the *de novo* production of T cells by the thymus (↑α) will reduce the time to reconstitute and will result in a highly diverse T-cell pool. Finally, a decrease in T-cell loss (↓d), e.g. longer cell lifespans, may help to accelerate T-cell reconstitution. We here investigated whether any of these possible homeostatic mechanisms occur in humans.

If there are indeed homeostatic mechanisms regulating lymphocyte dynamics when cell numbers are low, these should aid the recovery of T-cell numbers following lymphopenia. In theory, these mechanisms may take the form of increased T-cell proliferation, increased thymic output or decreased cell loss. Their effect on the speed of T-cell reconstitution and on the diversity of the recovered T-cell pool may be different (**Figure 1**). Increased peripheral proliferation of naive and memory T cells should lead to a faster restoration of homeostatic T-cell numbers but would not generate new T-cell specificities. Increased *de novo* T-cell production by the thymus, on the other hand, would not only help to speed up reconstitution but would also contribute to T-cell receptor (TCR) diversity in the peripheral T-cell pool. In theory, analysing alterations in TCR diversity during lymphopenia should thus provide information on the contribution of the thymus to immune reconstitution. Estimating the diversity of the T-cell repertoire when peripheral proliferation or T-cell loss are altered is not straightforward, however, because i) the impact of these dynamic parameters on T-cell diversity largely depends on whether or not all T-cell clones have equal chances to proliferate and survive, and ii) most TCR repertoire studies fail to distinguish between the loss of certain T-cell clones and the expansion of others (see **BOX 2**, T-cell receptor (TCR) repertoire analysis). The third potential response to lymphopenia would be decreased T-cell loss, e.g. due to reduced competition for survival factors. Although this should in principle help the reconstitution of the T-cell pool, in humans the effect may be limited. Naive T cells in humans, for example, have already very low death rates in normal circumstances (32, 33). Decreasing them even further may therefore have little effect on the speed of naive T-cell recovery. Although the relative roles of these potential homeostatic mechanisms may differ between different situations of lymphopenia, we consider all three of them when reviewing the evidence for homeostatic regulation of T-cell reconstitution in early development, healthy ageing, HIV infection, thymectomy and HSCT.

Box 2 **T-cell receptor (TCR) repertoire analysis.**

T-cell receptor (TCR) repertoire analysis following lymphopenia is a useful tool to study T-cell reconstitution. Despite great technical advances in high-throughput, next-generation sequencing (NGS), measuring the diversity of the T-cell compartment remains challenging (for in depth reviews on TCR repertoire analysis, see (28, 29)), because TCR repertoires are so diverse that any (blood) sample will inevitably contain only a very small fraction of the total T-cell repertoire and the translation of the information obtained from such small samples into estimates of total repertoire diversity is challenging (30, 31). An additional problem is that artefacts introduced during reverse transcription by PCR or during sequencing can mistakenly be interpreted as additional TCRs, while ignoring low frequency TCRs would lead to an underestimation of the number of true T-cell clones. Next to the remaining experimental difficulty to accurately determine TCR diversity, the interpretation of “*diversity*” measures is not unambiguous. While some measures of diversity, such as species richness, refer to the total number of different sequences within a sample, other measures, such as the Shannon or Simpson’s diversity indices, take the frequencies of different sequences into account. Although the latter indices may provide a more complete picture of the diversity of the T-cell pool, they may fail to distinguish between a lack of certain T-cell clones and the expansion of others, a distinction that is important when studying T-cell reconstitution following HSCT. Although estimates of the diversity of the human TCR repertoire thus need to be interpreted with caution, comparisons of TCR repertoire diversity, e.g. between patients and healthy controls, can provide useful information on the dynamics of T cells during immune reconstitution.

## Early development of the immune system

**Figure 2:**
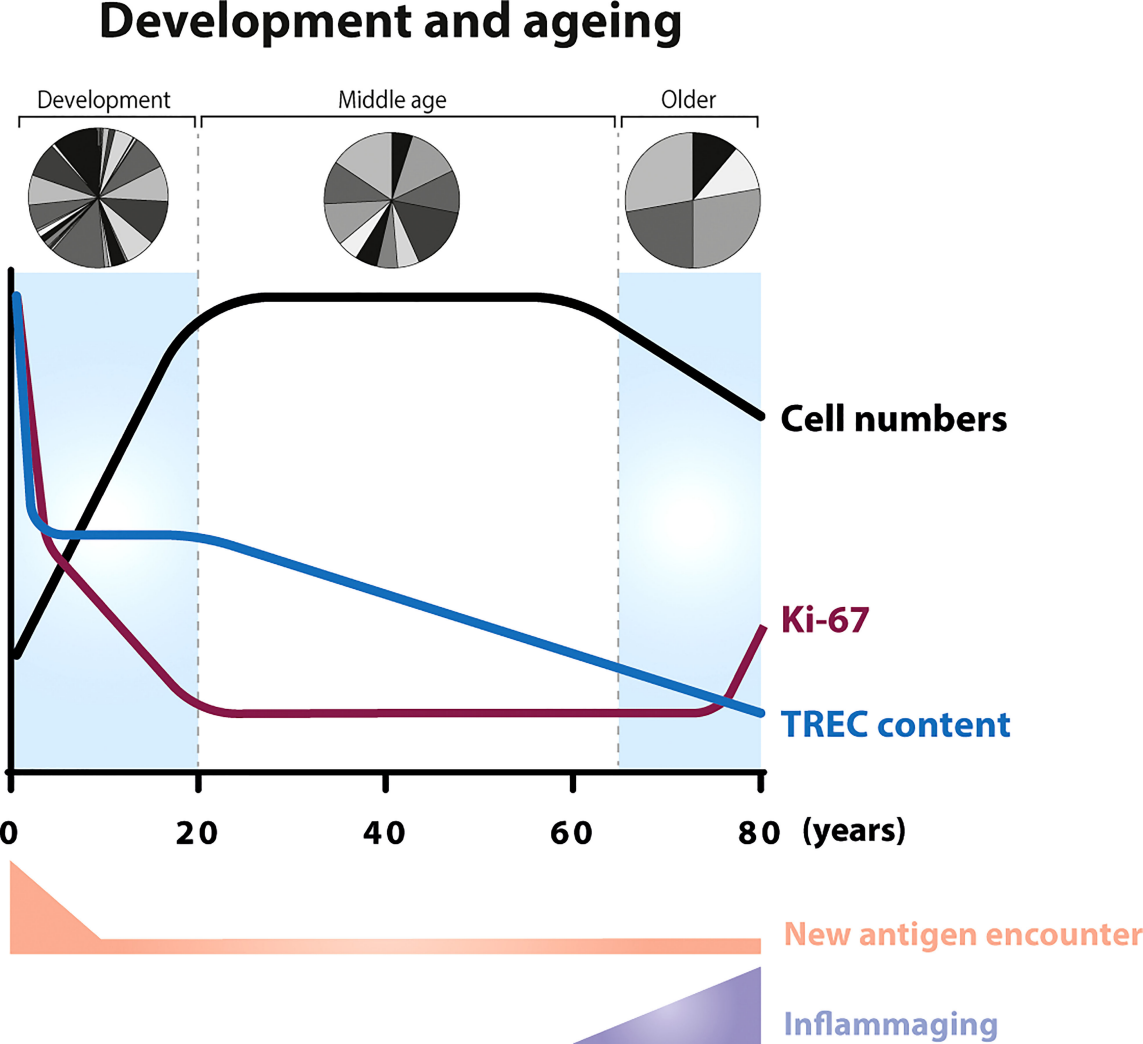
T-cell dynamics during ageing In neonates, the number of T cells increase as the body increases in size (34) (black line). The first weeks after birth, new-borns encounter a relatively large number of new antigens compared to later stages in life. In the foetus, the average T-cell receptor excision circle (TREC) content of CD4^+^ and CD8^+^ T cells (blue line) is relatively high (36) and, in new-borns, the majority of circulating T cells are naive (40). The fraction of proliferating T cells, as measured by Ki-67 expression, is 5-times higher in new-borns compared to adults (purple line). After the first half year of life, the percentage of Ki-67 positive T cells starts to decrease and stabilizes at adult age (36, 41, 42). Advanced age (above 70 years) is associated with a decline in T-cell numbers (43, 44), a reduced fraction of naive T cells (45), an increase in the percentage of Ki-67 positive cells (46, 47), and with persistent low level inflammation (inflammaging). With the gradual loss of thymopoiesis during ageing (33, 48), the TREC content decreases in an exponential fashion between the age of 20 and advanced age (13, 49). New-borns have been shown to have a greater repertoire diversity than young adults (39), while older individuals show skewing in the distribution of the clonal sizes (50).

Although the development of the human adaptive immune system begins in the foetus, at birth, the immune system is not yet fully developed, and it continues to mature until early infancy (34). Total body naive and memory T-cell numbers gradually expand from birth to 20 years of age. During foetal development and in the early post-natal period the majority of T cells are recent thymic emigrants (RTEs) (35); it is therefore perhaps not surprising that both the average T-cell receptor excision circle (TREC) content (36, 37), a commonly used measure of thymic output, and the level of diversity in the TCR repertoire (38, 39) are relatively high in neonates (**Figure 2**).

The fraction of proliferating lymphocytes in new-borns, as measured by Ki-67 expression, is significantly higher than in young adults, and declines during childhood (36, 41, 42) (**Figure 2**) (**BOX 3**, Ki-67 expression). Although at first sight, this seems to be suggestive of a cell density-dependent homeostatic mechanism, increased fractions of proliferating naive T cells were shown to be confined to the first half year of life (41), even though the total number of naive T cells expands until approximately 20 years of age. An alternative explanation for the high percentage of Ki-67 positive T cells in new-borns would be that increased peripheral T-cell proliferation is driven by the abrupt exposure of new-borns to new antigens, rather than by homeostatic mechanisms. However, the frequency of Ki-67^+^ T cells in cord blood from children born between 26 and 40 weeks of gestational age turned out to be highest in pre-term infants (36). This suggests that T-cell proliferation rates are even higher during foetal development and decrease over time even in the absence of new pathogen exposure. The latter study also showed that, despite the significant decrease in the fraction of Ki-67^+^ cells, TREC contents remained stable between 26 and 40 weeks of gestation (36). Because peripheral T-cell division should result in dilution of the average TREC content (**BOX 4**, How to measure thymic output?), the authors proposed that peripheral T-cell expansion and thymopoiesis in neonates complement each other, to ensure the generation of a diverse T-cell pool with optimal clone sizes (36). More recently, it was proposed that the increased levels of Ki-67 expression in the peripheral T-cell pool of young mice may reflect their proliferation status in the thymus rather than a lymphopenia-induced response to low T-cell numbers in the periphery during early development (66). Although the contribution of the thymus to the maintenance of the naive T-cell pool in adulthood is much smaller in humans than in mice (13), a similar explanation may underlie the increased levels of T-cell division in humans during their first years of life. Taken together, these data suggest that increased division rates, as measured by Ki-67 expression, of T cells from the peripheral blood may not always reflect a homeostatic response to help build up the T-cell pool, and may instead reflect a relatively recent thymic origin of the cells.

Box 3 **Ki-67 expression.**

Ki-67 expression is widely used as a marker of cell proliferation, both in basic research and in cancer diagnostics. Ki-67 is essential to spatially organise the heterochromatin and to control gene expression during cell division, by keeping chromosomes apart (51). Ki-67 protein levels and localisation vary throughout the cell cycle. The maximum expression of Ki-67 is found during mitosis (52), however its expression can be maintained for 4-5 days after the completion of cell division (53–55). Notably, even cells lacking Ki-67 can proliferate efficiently, which suggests that Ki-67 is not essential for cell division (56). Ki-67 expression data should therefore be interpreted with caution. First, under circumstances that may induce cell cycle arrest (e.g. under the influence of some HIV proteins) cells may keep on expressing Ki-67 while not completing their cell division (57). Second, Ki-67 may still be expressed during the G1 phase of the cell cycle, and the time cells spend in G1 may differ between cell types. Third, because the composition of the T-cell pool may be altered during lymphopenia, one may reach misleading conclusions about T-cell proliferation. For example, stem cell memory T cells (T_SCM_), which constitute a major proportion of the T-cell pool during the first weeks post-HSCT, present a naive-like phenotype and high levels of Ki-67 expression (35). Hence, if T_SCM_ cells are included in the “naive” T-cell population, it will lead to an increase in the fraction of Ki-67 positive “naive” T cells. Although this is not a specific drawback of Ki-67 data, one needs to take these issues into account when interpreting Ki-67 data. Finally, we and others recently observed that T cells isolated from blood are enriched for cells that express Ki-67 (58, 59), yet, higher Ki-67 expression in blood-derived T cells does not correspond to higher cellular turnover (58). We therefore suggest that Ki-67 can be used as an indicator of recent cell-cycle activity, but its expression is not a direct measure of cellular turnover.

Box 4 **How to measure thymic output?**

During T-cell receptor gene rearrangement, circular DNA excision fragments, so-called T-cell receptor excision circles (TRECs), are generated. TRECs are maintained episomally in the cell and are thought to be very stable over time **(A)**. The interpretation of TREC data is not straightforward (60). First, increased TREC content in a relatively empty T-cell pool does not *per se* reflect an increase in thymic output, and may in fact be the direct consequence of lymphopenia. With the same rate of thymic output, one new TREC-positive naive T cell will have a bigger contribution to the total TREC content in an empty naive T-cell pool than in a lymphoreplete pool (**B**, lower panel). Second, TREC levels are influenced by T-cell proliferation and death **(A)**. TRECs are not duplicated during mitosis, therefore T-cell proliferation results in the dilution of the average TREC content (61–64) (**C**, lower panel). Third, TREC measurements are usually performed in total PBMCs or bulk CD4^+^ and CD8^+^ T cells. The fraction of naive and memory T cells may differ between different groups of individuals especially during development and under lymphopenic conditions. A T-cell pool enriched in naive T cells, e.g. the neonatal T-cell compartment, will have a higher average TREC content than a T-cell pool with a higher fraction of memory T cells. As such, measurable TREC levels shortly after umbilical cord blood transplantation (<3 months) might be caused by the infusion of naive T cells present within cord blood grafts, which contain high TREC levels (65), rather than a reflection of actual thymic output. Combination of total TREC analysis within the naive T-cell population, T-cell proliferation, and expression of recent thymic emigrant markers on T cells, e.g. CD45RA, CCR7, CD62L, CD31, CD27, CD103, PTK7, CR2 and IL-8 expression (35), will provide further insights into the contribution of thymopoiesis to T-cell reconstitution.

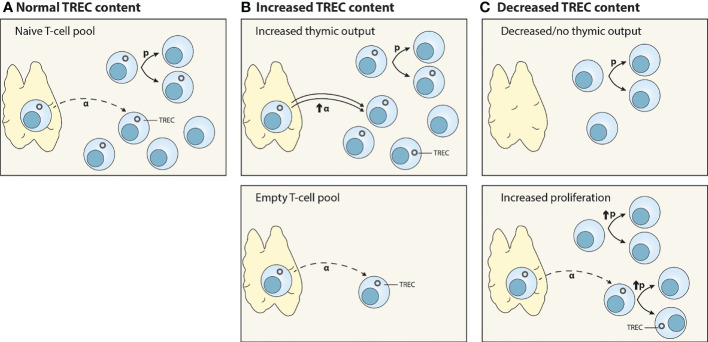

Interpretation of TREC data. **(A)** Normal thymic output (α) of TREC-positive T cells. TRECs are not duplicated during cell division; hence, T-cell proliferation (p) dilutes the average TREC content of a T-cell population. **(B)** Increased TREC content may be due to an increase in thymic output (α) (upper panel), or the consequence of normal thymic output into a relatively empty peripheral T-cell pool (lower panel). **(C)** Decreased TREC content may reflect decreased or no thymic function (upper panel), or increased peripheral proliferation (p) (lower panel).

## Healthy ageing

One of the most remarkable age-related changes of the immune system is the significant decline in thymic output in older individuals. In line with the originally estimated ten-fold decline in thymic output during adulthood (67), thymic output has been estimated to decrease from 16x10^6^ cells per day in young individuals to less than 1x10^6^ cells per day in older individuals (68). The frequency of double positive thymocytes in human thymic tissue decreases substantially after 40 years of age (69). The average TREC content and fraction of CD31^+^ (both markers of RTEs) T cells within the naive CD4^+^ T-cell pool also decline significantly with age (13, 35, 69). Furthermore, aging is thought to be associated with reduced TCR repertoire diversity of naive CD4^+^ and CD8^+^ T cells and memory CD4^+^ T cells, as measured by the Simpson’s index (50, 69), and by increased clonality of the naive T-cell pool (50) (**Figure 2**). This further supports that T-cell production *via* thymic output declines with age.

The decline in thymic output in older individuals is associated with reduced fractions of naive T cells and reduced absolute T-cell numbers (37, 43, 44, 69–71). Some studies have shown that this decline is accompanied by an increase in the fraction of Ki-67 positive naive CD4^+^ and CD8^+^ T cells (46, 47) (**Figure 2**). This led to the hypothesis that increased T-cell proliferation compensates for the relatively lymphopenic environment in older individuals. Ageing is, however, also associated with a low-grade persistent inflammatory status, a phenomenon termed inflammaging (72), which results in a continuous low level of T-cell activation. When using *in vivo* deuterium labelling to follow the dynamics of T cells in older individuals who had been selected for having a particularly good health status, we found that T-cell turnover rates were not increased in older individuals despite their significantly decreased naive T-cell numbers (33). These observations, together with the fact that increased T-cell proliferation is typically only observed after the age of 70 (46, 47) when the process of inflammaging is most predominant, suggest that increased levels of T-cell proliferation in older individuals are related to increased immune activation and may not reflect a homeostatic response to low cell numbers. They also demonstrate that increased T-cell division rates can easily be mistaken for a cell-density dependent response, if external factors driving T-cell division are not sufficiently taken into account.

## HIV infection

**Figure 3:**
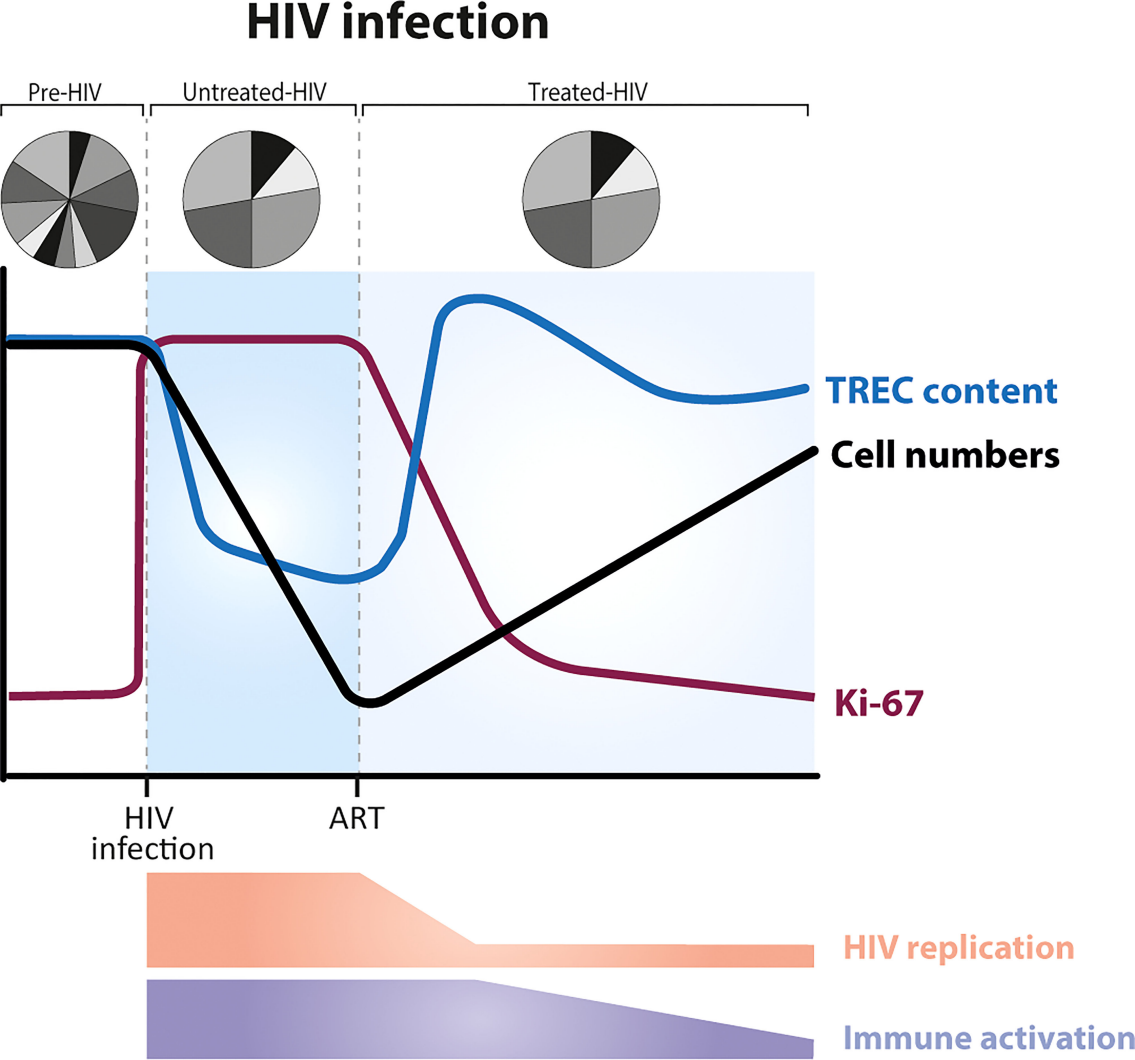
Dynamics of T cells during HIV infection, before and after the start of ART (anti-retroviral treatment). HIV infection is characterized by a severe and gradual depletion of CD4^+^ T cells (black line), which rise again after the start of ART (73). The fraction of Ki-67 positive CD4^+^ T cells (purple line) is increased during untreated HIV infection. ART leads to a rapid reduction of T-cell proliferation rates, long before T-cell numbers have recovered (61, 74). In most HIV-infected individuals, the average TREC content of CD4^+^ T cells is significantly decreased during untreated infection (due to increased T-cell proliferation) and increases on commencement of ART (75, 76) (blue line). TCR repertoire diversity is reduced, with a small proportion of TCRs highly expanded (77). Levels of immune activation and viral replication are high during untreated HIV infection, and decrease during ART.

HIV is one of the best-known examples of lymphopenia caused by pathogens. Untreated HIV infection is characterized by CD4^+^ T-cell lymphopenia, which becomes critical when CD4^+^ T-cell numbers are below 200cells/μl (73) (**Figure 3**). The dynamics of CD4^+^ T-cell reconstitution during antiretroviral treatment (ART) have been extensively studied. Following HIV infection, the fraction of Ki-67 positive cells increases and remains increased until the start of therapy. These increased levels of T-cell proliferation go hand in hand with significantly decreased T-cell TREC contents in untreated HIV infection - a natural consequence of the fact that TRECs are not copied during cell division (**BOX 4**, How to measure thymic output?). The fraction of proliferating CD4^+^ T cells in individuals with untreated HIV has been shown to inversely correlate with CD4^+^ T-cell numbers, suggesting that T cells proliferate faster to return to normal T-cell numbers (47, 78, 79). Yet, there is a large body of evidence suggesting that T-cell dynamics during HIV infection are to a large extent driven by the virus itself, and not necessarily by a homeostatic response to low T-cell numbers. HIV infection is a complex, multifactorial disease, in which, besides CD4^+^ T-cell lymphopenia, people present chronic immune activation associated with viral replication and bacterial translocation (80). In untreated individuals, the fraction of proliferating naive CD4^+^ T cells correlates with plasma viral load (47). Following ART, viral replication is effectively suppressed and the fraction of Ki-67 positive T cells quickly decreases, even when T-cell numbers are still low (61, 74). Moreover, based on *in vivo* deuterium labelling, we found that the turnover rates of naive CD4^+^ and CD8^+^ T cells in individuals who had been treated with ART for at least 1 year remained ~2-fold higher than in healthy individuals, even when total CD4^+^ T-cell numbers had already normalized (81). Together, these findings suggest that increased T-cell proliferation during HIV infection is mostly driven by the – in part perhaps irreversible – immunomodulatory effects of HIV infection, rather than by a homeostatic response to low CD4^+^ T-cell numbers.

Based on the observation that the average TREC content of CD4^+^ T cells increases during ART, concomitant with the recovery of naive CD4^+^ T-cell numbers (75, 76) (**Figure 3**), it has been proposed that, once viral replication is suppressed, increased thymic output plays a key role in the reconstitution of the T-cell pool (79). Computed tomography (CT) scans have shown that the adult thymus can indeed expand after the start of antiretroviral therapy (82–84). Nevertheless, these data fail to provide evidence for increased thymic function following ART (60, 85). Ongoing, but not necessarily increased, naive T-cell production by the thymus, and even low thymic production of new TREC^+^ naive T cells suffices to explain the observed increases in TREC content, given that total naive T-cell numbers are severely depleted (60, 85) (**BOX 4**, How to measure thymic output?). There is thus little evidence that during the lymphopenic state caused by HIV infection, either the output of T cells from the thymus or the rate of T-cell division are regulated in a cell density-dependent manner. More generally, these findings show that not only increased T-cell division rates but also increased TREC contents in a lymphopenic environment may not reflect a homeostatic response to low T-cell numbers.

## Thymectomy

Partial or total thymic tissue removal is routinely performed during cardiac surgery in neonates. Besides the study of lymphopenia, thymectomy provides a good framework to investigate the role of the thymus in humans. Following thymectomy, there is a significant reduction in naive T-cell numbers (65, 86–88). In most patients, CD4^+^ and CD8^+^ T-cell numbers have recovered to the level of age-matched healthy individuals approximately 10 years after neonatal thymectomy (65, 88).

In the first 5 years post-thymectomy, the fraction of Ki-67 positive, proliferating naive T cells is significantly (although not drastically) elevated compared with healthy children (47, 86, 88); thereafter, the percentage of proliferating naive T cells decreases to healthy control levels (88). The frequency of Ki-67 positive cells in thymectomyzed individuals has been shown to correlate inversely with the percentage and the absolute number of naive T cells (47), again suggestive for a homeostatic response to low T-cell numbers.

Interestingly, 5-10 years after thymectomy most children show signs of thymic tissue regrowth, based on CT and magnetic resonance imaging (MRI) scans and on the presence of RTEs in the peripheral T-cell pool, while a minority do not show any of these signs of thymic tissue regeneration (65, 88). In the latter children, total T-cell counts, the percentage of naive CD4^+^ T cells and total naive CD4^+^ T-cell numbers remain below normal ranges even 10 years post-thymectomy (65). The latter findings argue against the occurrence of lymphopenia-induced proliferation in the absence of thymic output, and suggest that thymopoiesis is essential for T-cell reconstitution. Future studies are needed to investigate whether T-cell proliferation rates are higher in thymectomized children who do not show signs of thymic tissue regeneration than in those who do.

## Lymphodepletion and HSCT

**Figure 4:**
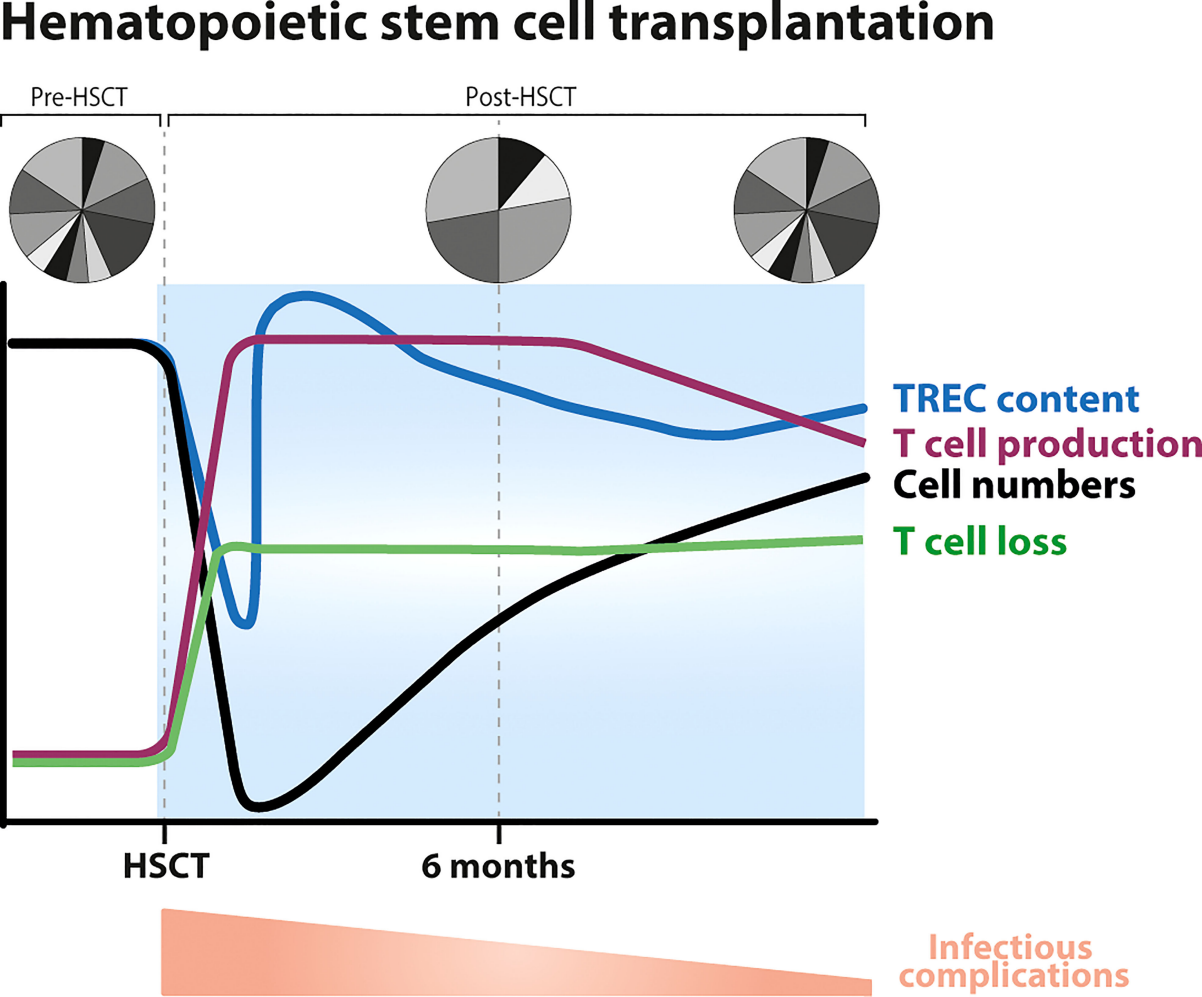
Dynamics of T-cell reconstitution following hematopoietic stem cell transplantation (HSCT). Following conditioning, T-cell numbers drastically decline (black line) after which they slowly recover, T-cell production levels increase (purple line), the average T-cell TREC content temporarily decreases (blue line) to then increase (possibly even reaching supra-normal levels) due to an influx of new naive T cells coming out of the thymus, and the TCR repertoire becomes less diverse due to clonal expansions (96, 97). CD8^+^ T cells typically reach normal levels within 3–6 months after HSCT, while the normalization of CD4^+^ T-cell numbers takes longer, sometimes even longer than 10 years. Total CD3^+^ T-cell numbers approach the normal range ~18 months after transplantation (19) (black line). T-cell production rates do not clearly correlate with T-cell reconstitution: they may decrease even when T-cell numbers have not yet completely recovered, and may stay higher than in healthy controls in subsets that have already recovered. Thymopoiesis is thought to be restored within 6 months to a year after HSCT, thereafter the TCR repertoire becomes more diverse and slowly normalizes. Full reconstitution of the T-cell pool is hampered by increased lymphocyte loss rates post-HSCT (19, 98–100). Post-HSCT there is a higher occurrence of infectious complications, which gradually normalizes.

Of all the different situations in humans that cause some degree of lymphopenia, HSCT and the accompanying requirement for severe conditioning regimens perhaps pose the biggest challenge on immune recovery and may thus provide the best setting to find signs of a homeostatic response to low T-cell numbers. Myeloablative conditioning regimens are known to affect thymopoiesis and significantly delay T-cell reconstitution (89, 90). Using thoracic CT scans it has been shown that soft thymic tissue is significantly reduced at the end of the conditioning regimen, and that thymic mass regenerates to pre-transplant levels approximately one-year post-HSCT (49). By quantifying absolute numbers of CD31^+^ naive CD4^+^ T cells and Sj/β TREC ratios, it has been shown that RTEs are reduced in numbers shortly after lymphodepletion (91) and that they start to rise approximately 6 months post-HSCT (90, 92–94), especially in young individuals, but also in older recipients (95) (**Figure 4**). The average TREC content of peripheral blood mononuclear cells has been shown to be increased for at least 2 years post-HSCT (101, 102) (**Figure 4**). The latter observation has been interpreted as evidence for increased thymic output in HSCT patients compared to healthy individuals (101). However, as mentioned before, increased TREC contents post-lymphopenia may in fact be a direct consequence of a depleted T-cell pool (60) (**BOX 4**, How to measure thymic output?). Although high average TREC contents during immune reconstitution provide evidence that the thymus is exporting new naive T cells, this observation does not *per se* imply that thymic output in HSCT patients is increased compared to healthy individuals.

Independent studies quite convincingly show that T-cell proliferation rates are increased in patients undergoing HSCT. Indeed, the fraction of Ki-67 positive, proliferating naive and memory CD4^+^ and CD8^+^ T cells has been shown to be increased during the first 3 months post-alemtuzumab, post-ATG treatment and post-HSCT (18, 19, 91, 103, 104). Notably, as soon as 3-6 months post-treatment, the fraction of Ki-67 positive T cells decreases, while patients are still severely T-cell lymphopenic (18, 103–105) (**Figure 4**). The fact that T-cell proliferation normalizes when patients are still lymphodepleted and that T-cell proliferation correlates with the occurrence of clinical events (e.g. GVHD or infections) and not with T-cell numbers (105) suggest that increased T-cell proliferation in many patients is in fact triggered by lymphopenia-related and HSCT-related complications rather than by low T-cell numbers. To try and avoid the influence of such confounding factors, we have studied T-cell dynamics by *in vivo* deuterium labelling in patients who received an autologous HSCT and had no signs of clinically manifested infections or GHVD. We found that the production rates of most T-cell subsets in these patients were 2-8 fold increased as compared to healthy age-matched controls (106). Although this was suggestive of a lymphopenia-induced proliferative response in these patients, we − rather unexpectedly − observed that these rates remained increased in the few T-cell subsets of which cell numbers had already normalized (106).

In the same study, we also estimated T-cell loss rates, and much to our surprise found that increased T-cell production rates in these patients went hand in hand with a 3-fold increase in T-cell loss rates, even for T-cell subsets that had far from normalized in cell numbers (106). Again, this would not be expected if T-cell loss rates were homeostatically regulated like they are in mice. Unfortunately, deuterium labelling cannot distinguish between T-cell death and loss of T cells from the population of interest through migration or differentiation. Based on these data alone we can therefore not conclude that cells died more quickly in patients who had undergone HSCT. Alternatively, they may have undergone increased differentiation or migration out of the blood. Some studies have reported that T-cell death, preferentially of activated cells, is increased post-HSCT, thereby impairing T-cell reconstitution (19, 98–100). It has been shown that following HSCT there is an increase in the fraction of apoptotic, 7-AAD (7-amino-actinomycin D) positive T cells (100) and in the expression of CD95 (98) by T cells, and a reduction in the expression of interleukin-7 receptor α (IL-7Rα, CD127) and the anti-apoptotic protein Bcl-2 (98, 99). Together, these findings suggest that T-cell death rates post-HSCT are increased during lymphopenia, and not regulated based on cell densities.

As mentioned before, T-cell deficiency post-HSCT, post-alemtuzumab and post-ATG treatments is associated with an increased incidence of clinical and sub-clinical conditions, viral reactivation, opportunistic infections and GVHD (24, 107, 108). CMV seropositivity and reactivation turn out to play a major role in T-cell reconstitution, in that reconstitution is quicker in CMV seropositive patients and in patients in whom CMV reactivation occurs, largely due to a rapid recovery of effector memory T cells and a progressive accumulation of CMV-specific CD8^+^ T cells (96, 103). Fast T-cell recovery and high T-cell proliferation post-HSCT may thus not reflect a homeostatic compensation for low lymphocyte numbers, and may even yield a T-cell repertoire that is heavily skewed toward recognition of chronic infections.

## Concluding remarks

Based on experiments in mice, the recovery of T cells following lymphopenia is generally thought to be homeostatically regulated in a cell density-dependent manner. In all circumstances of lymphopenia in humans that we reviewed here, we found no unambiguous evidence for increased thymic output or reduced cell death during lymphopenia. The only change that was observed in several lymphopenic circumstances is an increase in T-cell proliferation rates. Although increased T-cell proliferation rates are generally interpreted as a homeostatic mechanism to compensate for low T-cell numbers, we found no unequivocal evidence that these changes in cell proliferation reflect a response to low cell numbers. Instead, increased proliferation rates in lymphopenic humans may equally well reflect T-cell expansion triggered by inflammation, infection, GVHD, therapy-related tissue damage, or exposure to new antigens. Because T-cell depletion in humans goes hand in hand with lymphopenia- and disease-related complications, future studies are needed to disentangle true homeostatic regulation from the effects of immune activation. This should extend to different causes of T-cell depletion in humans, since each condition may be unique and the dynamics of T-cell reconstitution may not be the same in all situations.

## Author contributions

All authors contributed to the article and approved the submitted version.

## Funding

This work was supported by funding from the European Union Seventh Framework Programme (FP7/2007-2013) through the Marie-Curie Action “Quantitative T Cell Immunology” Initial Training Network, with reference FP7-PEOPLE-2012-ITN 317040-QuanTI supporting MB-P.

## Conflict of interest

The authors declare that the research was conducted in the absence of any commercial or financial relationships that could be construed as a potential conflict of interest.

## Publisher’s note

All claims expressed in this article are solely those of the authors and do not necessarily represent those of their affiliated organizations, or those of the publisher, the editors and the reviewers. Any product that may be evaluated in this article, or claim that may be made by its manufacturer, is not guaranteed or endorsed by the publisher.
